# Pediatric data of the usefulness of estimated average glucose (eAG) in glycemic control and cardiovascular risk reduction

**DOI:** 10.1016/j.dib.2020.105993

**Published:** 2020-07-05

**Authors:** Dandan Wang, Yu Chen

**Affiliations:** a Department of Laboratory Medicine, Dr. Everett Chalmers Regional Hospital, Horizon Health Network, Fredericton, NB, Canada; b Department of Laboratory Medicine, Shengjing Hospital of China Medical University, Shenyang, Liaoning, China; c Department of Pathology, Dalhousie University, Halifax, NS, Canada

**Keywords:** Estimated average glucose, Glycemic control, Cardiovascular risk, Pediatrics, Diabetes

## Abstract

The Estimated Average Glucose (eAG) is assumed to provide patients a better understanding of their recent average blood sugar levels comparing to HbA1c, therefore better control their glycemic levels. However, since its inception, debates on its clinical utility have been over several years leading to an unpopular laboratory and clinical practice of adoption; and there is no evidence to support or against the usefulness of eAG in real world medical practice.

Data set presented in this article is related to our research paper entitled “Usefulness of Estimated Average Glucose (eAG) in glycemic Control and Cardiovascular Risk Reduction”, available in Clinical Biochemistry [1]. In this article, we compared population lipid and glycemic controls in pediatric diabetic patients of the regional health authority (RHA) zone 1.1 in New Brunswick, Canada, before and after the eAG implementation in January 2010, and with other 7 zones that do not report the parameter. Data (7,355 HbA1c values and 2,062 LDL-c values) was extracted from all pediatric diabetic patients in the Provincial Diabetes Registry from 2008 to 2014. The proportions of patients achieving therapeutic targets (HbA1c<53 mmol/mol (7.0%) and LDL-*c*<2.6 mmol/L) and the distributions of HbA1c and LDL-c values pre/post the eAG implementation in RHA Zone 1.1 were assessed. Additionally, to investigate whether the glycemic and cholesterol control in pediatric diabetic patients in RHA Zone 1.1 after the implementation of eAG was better than in other zones, we also compared the medians and inter quartile ranges of HbA1c and LDL-c from different zones from 2010 to 2014.

Specifications table

**Table:** 

Subject	Clinical Biochemistry
Specific subject area	Endocrinology, Diabetes and Metabolism, Lipid metabolism, Pediatric data
Type of data	Figure
How data were acquired	This retrospective study was conducted by extracting HbA1c and LDL-c data for all zones from the New Brunswick Diabetes Registry, anonymously.
Data format	Raw
	Analyzed
Parameters for data collection	The New Brunswick Diabetes Registry identifies diabetes based on the Canadian Chronic Disease Surveillance System (CCDSS) and HbA1c data. The diabetes registry includes patients meeting one of the following criteria: (a) Two Medicare claims within a 2 year period that specify diabetes as the reason for the visit, (b) A hospital admission (from Discharge Abstract Data) with a diagnosis of diabetes, whether or not it was the primary reason for admission, (c) A historical record of at least one HbA1c ≥ 48 mmol/mol (6.5%).
Description of data collection	Data for pediatric diabetic patients were obtained from the New Brunswick Diabetes Registry from 2008 to 2014.
Data source location	Fredericton, New Brunswick, Canada
Data accessibility	With the article
Related research article	Wang D, Chen Y, Usefulness of Estimated Average Glucose (eAG) in Glycemic Control and Cardiovascular Risk Reduction, Clin. Biochem. (EPub on June 14, 2020). doi: 10.1016/j.clinbiochem.2020.06.009.

## Value of the data

Value of the Data

• This study provides previously unreported the clinical usefulness of estimated average glucose (eAG) on glycemic control and blood lipid control in *pediatric* diabetic patients.
• The data helps clinicians and medical laboratories to decide on whether to implement eAG calculation or not.
• On a national and international level, these data can assist as a basis for further studies on diabetic biomarker evaluation and assessment.

## 1 Data description

**Fig. 1:**
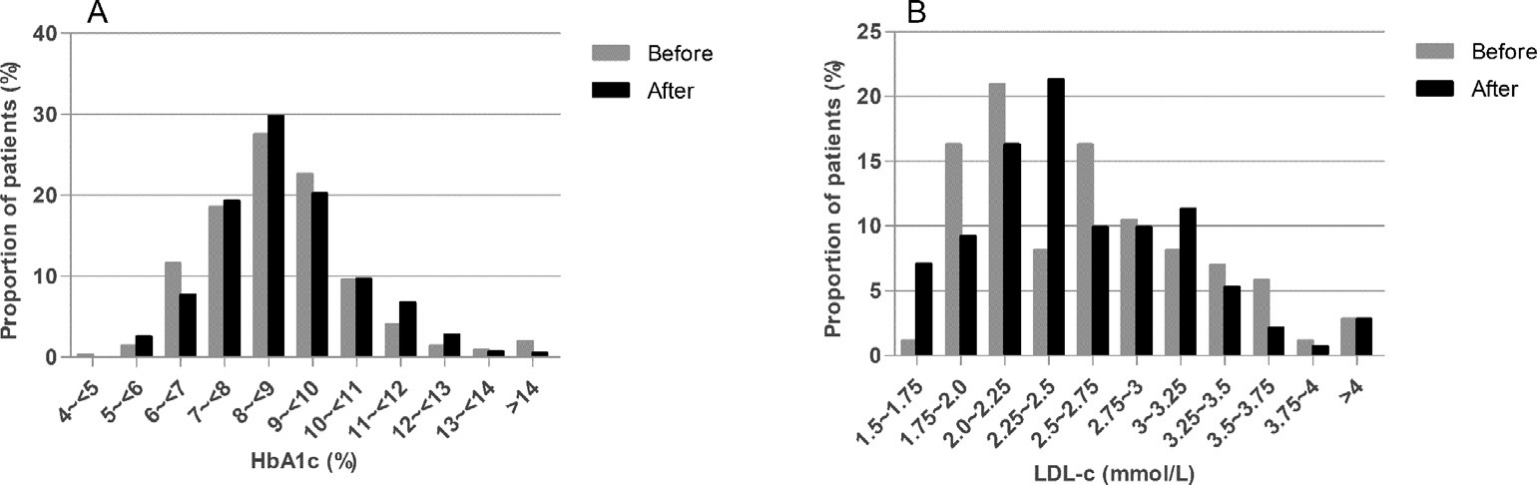
Distributions of HbA1c and LDL-c values of pediatric diabetic patients in Zone 1.1 before and after eAG implementation. A, HbA1c values; B, LDL-c values.

**Fig. 2:**
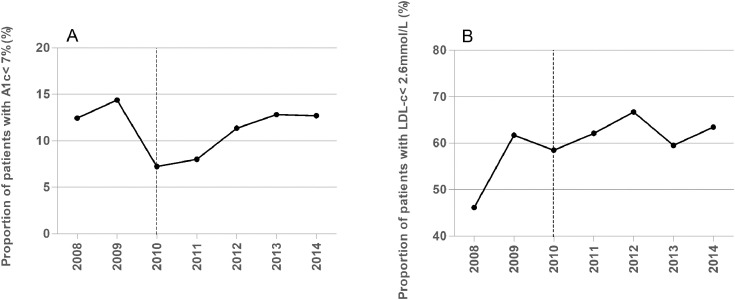
Proportions of pediatric diabetic patients achieving therapeutic targets (HbA1c<53 mmol/mol (7.0%) and LDL-*c*<2.6 mmol/L) in Zone 1.1 before and after eAG reporting. A, HbA1c values; B, LDL-c values. Dashed vertical line indicates when Zone 1.1 started to report eAG (January 2010).

**Fig. 3:**
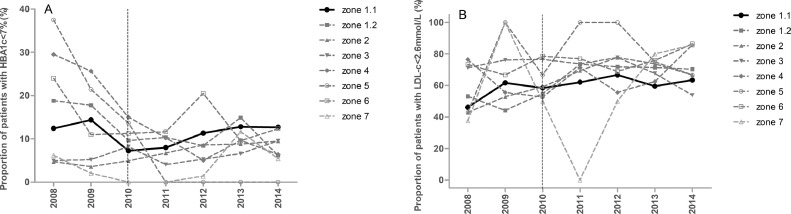
Proportions of pediatric diabetic patients achieving therapeutic targets of HbA1c and LDL-c in all 8 regional health authority zones of New Brunswick from 2008 to 2014. Of these zones, only Zone 1.1 reported eAG. A, HbA1c values; B, LDL-c values. Dashed vertical line indicates when Zone 1.1 started to report eAG (January 2010).

**Fig. 4:**
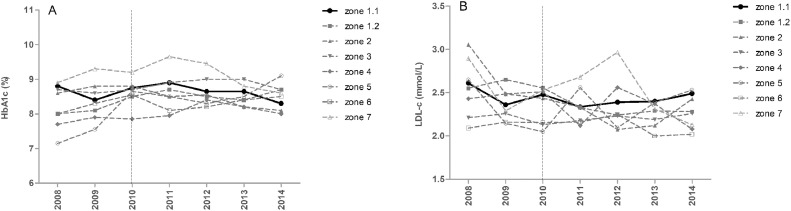
Overall medians of HbA1c and LDL-c values of pediatric diabetic patients in all 8 regional health authority zones of New Brunswick from 2008 to 2014. Of these zones, only Zone 1.1 reported eAG. A, HbA1c values; B, LDL-c values. Dashed vertical line indicates when Zone 1.1 started to report eAG (January 2010).

**Fig. 5:**
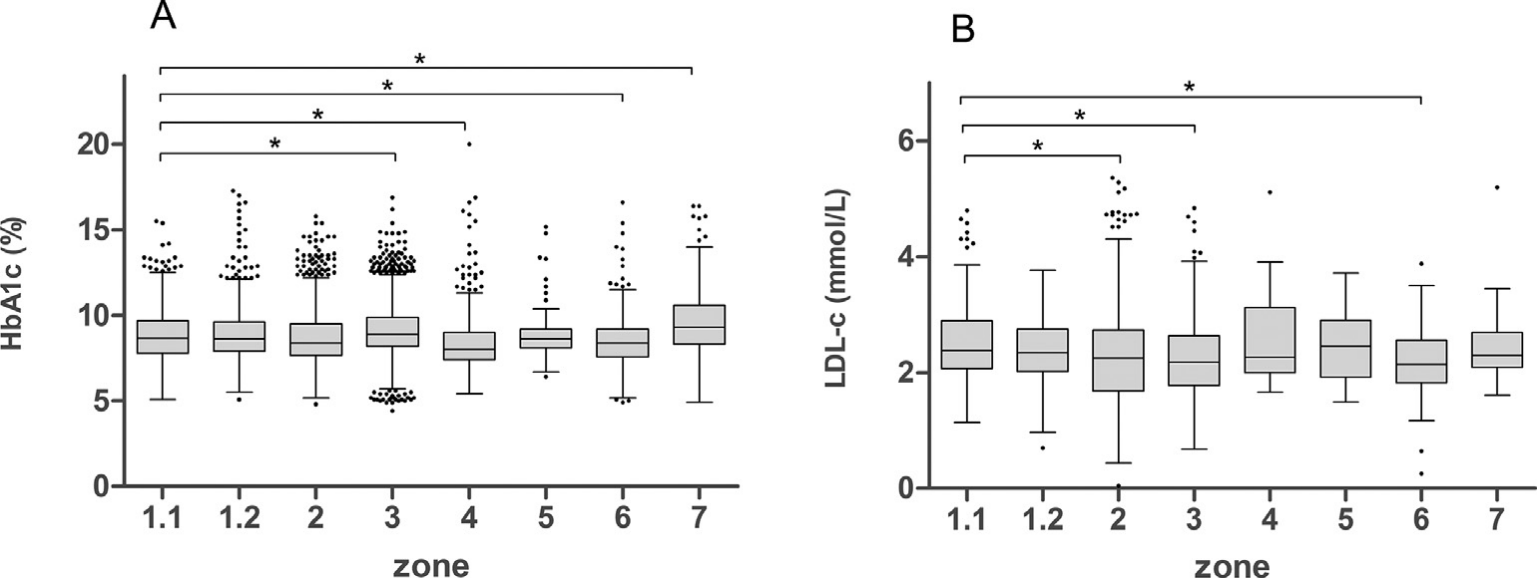
Distributions of HbA1c (A) and LDL-c (B) values of pediatric diabetic patients in 8 regional health authority zones of New Brunswick from 2010 to 2014. The endpoint of upper whisker is the 75th percentile plus 1.5 times IQR, the endpoint of lower whisker is the 25th percentile minus 1.5 times IQR, the line in the box shows the median, the lower and upper edges of box correspond to the first and third quartiles. *Significant difference (*p* < 0.05), compared with Zone 1.1.

The data contains information on the distributions of HbA1c and LDL-c values (Fig. 1), and proportions of diabetic patients achieving therapeutic targets (HbA1c<53 mmol/mol (7.0%) and LDL-*c*<2.6 mmol/L) (Fig. 2) of pediatric diabetic patients in Zone 1.1 before and after eAG implementation in January 2010. It also contains information on the proportions of diabetic patients achieving therapeutic targets of HbA1c and LDL-c (Fig. 3) and overall medians of HbA1c and LDL-c values (Fig. 4) in all 8 regional health authority zones of New Brunswick from 2008 to 2014. Furthermore, the distributions of HbA1c (A) and LDL-c (B) values of pediatric diabetic patients in all 8 regional health authority zones of New Brunswick from 2010 to 2014 has been demonstrated in Fig. 5. The raw data is also provided as a supplementary file.

## 2 Experimental design, materials and methods

### 2.1 Subjects

This retrospective study was conducted by extracting HbA1c and LDL-c data for all zones from the New Brunswick Diabetes Registry, anonymously, follow the Department of Health privacy and confidentiality policies. There were 8 RHA zones in New Brunswick, among these zones, only Zone 1.1 has been reporting eAG since January 2010. There were total 9417 data points, consisting of 7355 HbA1c values and 2062 LDL-c values from 2008 to 2014. Of these data, 4798 were from male, aged between 3 and 17; and 4619 were from female, aged between 1 and 17. The study was approved by the Research Ethics Board of Horizon Health Network (File#2015–2087). The Research Ethics Board for Horizon Health Network is organized and operates according to the principles of the Harmonized Tripartite Guidelines: Good Clinical Practice, Tri-Council (Canadian Institutes of Health Research, Natural Sciences and Engineering Research Council of Canada, and Social Sciences and Humanities Research Council of Canada) Policy Statement and Division 5 of the Food and Drug Regulations.

### 2.2 Data analysis

One of the main evaluation indicators we took to assess the utility of eAG was the overall proportions of patients achieving therapeutic targets, i.e. HbA1c<53 mmol/mol (7.0%) and LDL-*c* <2.6 mmol/L as per American Diabetes Association guidance document [2]. The distributions of HbA1c and LDL-C levels in Zone 1.1 between 2008 and 2009 as the baseline and 2010–2014 after reporting eAG were analyzed. We also compared the proportions of diabetic patients achieving therapeutic targets in Zone 1.1 before and after the eAG was implemented.

Additionally, to compare whether the glycemic and cholesterol control in diabetic patients in Zone 1.1 after the implementation of eAG was better than those of other zones, the proportions of patients achieving therapeutic targets and the overall medians of HbA1c and LDL-c in each RHA zones were analyzed.

The Kruskal-Wallis statistic was conducted to compare the medians and inter quartile ranges of HbA1c and LDL-c from different zones from 2010 to 2014. Zone 1.1 was statistically different (*p* < 0.05) comparing with Zones 3, Zone 4, Zone 6 and Zone 7 on HbA1c; Zone 1.1 was statistically different (*p* < 0.05) comparing with Zones 2, Zone 3, and Zone 6 on LDL-C. Overall, the glycemic control and blood lipid control in pediatric diabetes patients in Zone 1.1 was at an intermediate level in all 8 zones.

## Declaration of Competing Interest

The authors declare that they have no known competing financial interests or personal relationships which have, or could be perceived to have, influenced the work reported in this article.

## Appendix Supplementary materials

Supplementary material 1. Pediatric raw data for HbA1c and LDL-c. Image, application 1
